# Clinical Patterns and Outcome of Hepatocellular Carcinoma in Patients with Nonalcoholic Fatty Liver Disease

**DOI:** 10.1155/2020/4873875

**Published:** 2020-06-03

**Authors:** Seon Young Ahn, Suk Bae Kim, Il Han Song

**Affiliations:** Division of Hepatology, Department of Internal Medicine, Dankook University College of Medicine, Dankook University Hospital, 201 Manghyang-ro, Dongnam-gu, Cheonan, Chungcheongnam-do 31116, Republic of Korea

## Abstract

**Background:**

Nonalcoholic fatty liver disease (NAFLD) may develop into liver cirrhosis and hepatocellular carcinoma (HCC). The aim of this study was to compare the clinical patterns and survival outcomes of NAFLD-related HCC patients and those of alcoholic liver disease (ALD)-related or hepatitis B virus (HBV)-related HCC patients.

**Methods:**

A total of 622 HCC patients with associated NAFLD (*n* = 56), ALD (*n* = 173), or HBV infection (*n* = 393) were enrolled. The clinical characteristics and survival were analyzed according to the underlying liver diseases.

**Results:**

NAFLD-related HCC patients were more commonly older women and had more metabolic risk factors but were less likely to have cirrhosis and ascites, compared to ALD-related or HBV-related HCC patients. NAFLD-related HCC more often had an infiltrative pattern (*P*=0.047), a larger tumor (*P*=0.001), more macrovascular invasion (*P*=0.022), and exceeded the Milan criteria (*P*=0.001), but was less frequently diagnosed during tumor surveillance (*P*=0.025). Survival analysis did not show any difference among NAFLD-related, ALD-related, and HBV-related HCC patients. Furthermore, propensity score matching analysis did not reveal a significant difference in the median survival between the different groups (NAFLD vs. ALD, 14.0 months [95% confidence interval (CI), 2.0–26.0] vs. 13.0 months [95% CI, 0–26.3]; *P*=0.667, NAFLD vs. HBV, 14.0 months [95% CI, 2.0–26.0] vs. 12.0 months [95% CI, 4.3–17.8]; *P*=0.573).

**Conclusions:**

NAFLD-related HCCs were more often detected at an advanced stage with infiltrative patterns, although they showed no significant difference in survival compared to ALD-related or HBV-related HCCs. A future prospective research should be focused on identifying NAFLD patients who require strict surveillance in order to early detect and timely treat HCC.

## 1. Introduction

Liver cancer is the sixth most common solid tumor with over half a million new cases and the second leading cause of cancer death worldwide [1]. Hepatocellular carcinoma (HCC) accounts for 70% to 80% of the total liver cancer burden, representing the major histological subtype of primary liver malignancies [2]. HCC is frequently associated with fibrotic or cirrhotic liver disease and is mainly due to hepatitis B virus (HBV), hepatitis C virus (HCV), and alcohol abuse [3, 4]. The incidence of HCC has been increasing over the last two decades in several developed countries including the United States and Japan, as well as in Europe [5–7]. Approximately 50% of the new cases are owing to the large number of patients with chronic hepatitis C, while chronic hepatitis B and alcoholic liver disease (ALD) were other contributing factors [7, 8]. However, the etiology of HCC in 15% to 50% of new HCC cases remains unclear, suggesting that other risk factors are responsible for the observed increase in the incidence of HCC [9, 10]. In recent years, nonalcoholic fatty liver disease (NAFLD) was suggested to be the cause of disease in a large number of these cases with unknown etiology [10–13]. NAFLD is characterized by excessive accumulation of lipids within the cytoplasm of hepatocytes in people who do not consume alcohol; it encompasses a broad spectrum of features, ranging from simple reversible steatosis to the presence of inflammation and/or fibrosis, which can progress to cirrhosis and HCC [14]. In addition, NAFLD represents a hepatic manifestation of metabolic syndrome, and its prevalence is rapidly increasing along with the increase in obesity and type 2 diabetes mellitus [15, 16]. Based on the prevalence and natural history of NAFLD, it may actually be the primary cause of HCC [13, 17, 18]. However, few studies have compared the clinical patterns and outcomes of HCC patients according to the etiologies of HCC. Therefore, this study aimed to compare the clinical features and survival outcomes of NAFLD-related HCC patients and those of ALD-related or HBV-related HCC patients.

## 2. Methods

### 2.1. Study Design and Subjects

**Figure 1:**
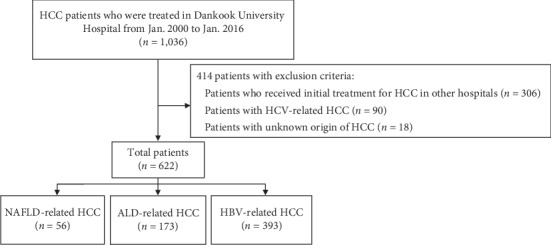
Schematic diagram of the study design and patients enrollment. HCC: hepatocellular carcinoma; ALD: alcoholic liver disease; HBV: hepatitis B virus; NAFLD: nonalcoholic fatty liver disease; HCV: hepatitis C virus.

This was a retrospective, comparative, observational study of HCC patients who were treated in Dankook University Hospital between January 2000 and January 2016. After reviewing the medical records of 1,036 HCC patients, we excluded patients who had undergone initial treatment in other hospitals (306 patients), patients with HCV-related HCC (90 patients), and patients with unknown origin of HCC (18 patients). A total of 622 HCC patients with liver issues associated with NAFLD (56 patients), ALD (173 patients), or chronic HBV infection (393 patients) who underwent initial treatment at OO University Hospital were enrolled in the present study (Figure 1).

### 2.2. Disease Diagnosis and Definition

HCC was diagnosed histologically or clinically according to the 2018 Practice Guidance by the American Association for the Study of Liver Diseases (AASLD) [19]. HCC surveillance was defined as the repeated application of screening tools with alpha-fetoprotein and ultrasound at a 6-month interval for patients at high risk. Liver cirrhosis was diagnosed based on histologic, radiologic, biochemical, and/or endoscopic evaluation. The diagnosis of ALD was made if the patient had a history of significant alcohol consumption with clinical evidence of liver disease and corresponding laboratory abnormalities [20]. Significant alcohol consumption was defined as an alcohol intake exceeding 30 g (approximately half a bottle of soju)/day in males and 20 g/day in females for at least 10 years. Chronic HBV infection was defined as positivity for hepatitis B surface antigen (HBsAg) and positive nucleic acid test results on two occasions for HBV DNA including qualitative, quantitative, and genotype testing, at least 6 months apart, regardless of the serostatus of hepatitis B e antigen (HBeAg) [21]. Patients were classified as having NAFLD if hepatic steatosis was evident on histology or radiology and if all other known causes of secondary hepatic fat accumulation could be ruled out, including significant alcohol consumption, use of lipogenic medications, and hereditary liver disorders [22]. Hepatic steatosis was radiologically diagnosed if the ultrasound image showed a diffusely increased echogenicity of the liver parenchyma which is clearly brighter than the renal cortex and computerized tomography image showed hepatic attenuation, evaluated as Hounsfield units, much lower than that of the spleen. Hypertension was defined as a blood pressure ≥ 140/90 mmHg or ongoing antihypertensive treatment, and diabetes was considered as fasting serum glucose ≥ 130, hemoglobin A1c ≥ 6.5%, or ongoing antidiabetic treatment.

### 2.3. Clinical Data Analysis

At the time of the first HCC diagnosis, the following data were recorded: demographic variables (age, gender, and body mass index), metabolic risk factors (prediabetes/diabetes, hypertension, and dyslipidemia), liver function test results, detection pattern of the tumor, tumor characteristics, and type of treatment. The treatment performed at the time of entry into the study was the first treatment. Survival was analyzed according to the etiology of the underlying liver disease. Treatment was selected according to the current guidelines after considering the clinical, biochemical, and oncologic characteristics of patients. When no oncologic treatment was administered, treatment was categorized as “best supportive care.” Patients who were lost to follow-up were censored at the last time they were examined.

### 2.4. Statistical Analysis

Statistical analysis was performed by using SPSS version 20.0 for Windows (SPSS. Inc., Chicago. IL, USA). Continuous variables were expressed as the mean and standard deviation or median and interquartile range after adjusting for normal distribution and were compared using the Student's *t*-test. Categorical variables were expressed as the number of cases and proportions and were compared using the Fisher's exact test. Across-group comparisons of quantitative variables were performed with analysis of variance (ANOVA). Survival was measured as the interval between the time of HCC diagnosis at Dankook University Hospital and the time of the last follow-up or death; it was analyzed and compared using the Kaplan–Meier method with the log-rank test. To reduce bias due to confounding variables, we performed propensity score matching analysis while considering the main variables that have a clinically known impact on survival and that show significant differences between NAFLD-related and ALD-related HCC patients or NAFLD-related and HBV-related HCC patients. This propensity model was used to perform one-to-one matching using the nearest-neighbor matching method, in which the matching variables, such as age, gender, Child-Pugh classification, tumor characteristics, and type of treatment were entered. A *P* value < 0.05 was considered statistically significant.

## 3. Results

### 3.1. Clinical Characteristics

The baseline demographic and clinical characteristics of patients are shown in Table 1. NAFLD-related HCC patients were significantly older (68.0 ± 10.9 years vs. 64.1 ± 9.4 years, 56.2 ± 10.4 years; *P* < 0.001), less commonly men (62.5% vs. 96.5%, 79.4%; *P* < 0.001), and more frequently had metabolic risk factors that included diabetes mellitus (48.2% vs. 33.5%, 19.6%; *P* < 0.001) and hypertension (42.9% vs. 27.2%, 21.9%; *P*=0.003) compared to ALD-related or HBV-related HCC patients. Liver cirrhosis (75% vs. 93.1%, 90.6%; *P* < 0.001) and ascites (32.1% vs. 56.1%, 47.1%; *P*=0.006) were also less common, and Child-Pugh score (6.2 ± 1.4 vs. 6.9 ± 1.6, 6.7 ± 1.8; *P*=0.014) and Model for End-Stage Liver Disease (MELD) score (9.1 ± 4.1 vs. 12.0 ± 7.3, 11.3 ± 6.9; *P*=0.021) were slightly lower in NAFLD-related HCC patients than in the other two groups.

### 3.2. Tumor Characteristics

The tumor characteristics of patients are shown in Table 2. NAFLD-related HCCs were diagnosed less frequently during tumor surveillance (76.8% vs. 85.5%, 89.6%; *P*=0.025) compared to ALD-related or HBV-related HCCs. In addition, NAFLD-related HCCs had a larger tumor (mean diameter 6.2 ± 3.4 cm vs. 3.7 ± 3.6 cm, 4.5 ± 4.0 cm; *P*=0.001), more often had an infiltrative pattern (26.8% vs. 13.3%, 15.0%; *P*=0.047) and macrovascular invasion (30.4% vs. 19.1%, 30.3%; *P*=0.022), and exceeded the Milan criteria (62.5% vs. 35.8%, 46.8%; *P*=0.001) compared to the other two groups. Barcelona Clinic Liver Cancer (BCLC) stage 0 (5.4% vs. 13.9%, 17.8%; *P*=0.043) or A (32.1% vs. 43.9%, 32.6%; *P*=0.029) was significantly less common while BCLC stage C (35.7% vs. 12.7%, 23.2%; *P* < 0.001), which is more advanced disease, was more frequent in NAFLD-related HCC patients than in other two groups. The median levels of alpha-fetoprotein (182 ng/dL vs. 26.8 ng/dL, 70.5 ng/dL; *P* < 0.001) and protein induced by vitamin K absence II (PIVKAII) (654.5 mAU/mL vs. 94.5 mAU/mL, 45.5 mAU/mL; *P* < 0.003 were also higher in NAFLD-related HCC patients than in the other two groups. Different patterns of clinical and tumor characteristics led to slightly different treatment strategy in the three groups (Table 3). Transcatheter arterial chemoembolization (50.0% vs. 64.7%, 55%; *P*=0.027) was less commonly used in NAFLD-related HCC patients. More patients with NAFLD-related HCC compared to the other two groups were eligible for liver resection (19.6% vs. 6.9%, 14.2%; *P*=0.014) and underwent sorafenib treatment (16.1% vs. 3.5%, 9.4%; *P*=0.006).

### 3.3. Survival Outcomes

**Figure 2:**
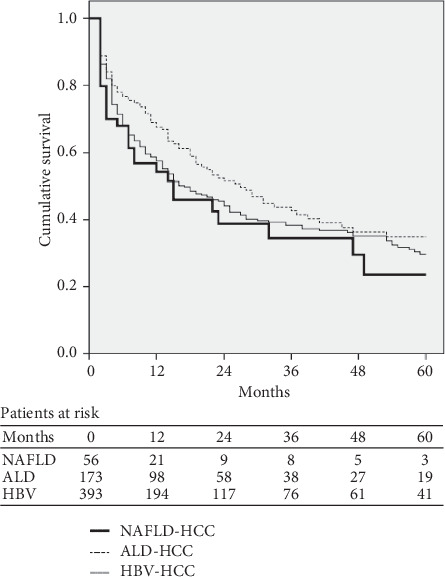
Survival curves of patients with HCC according to underlying etiologies. ALD: alcoholic liver disease; HBV: hepatitis B virus; HCC: hepatocellular carcinoma; NAFLD: nonalcoholic fatty liver disease.

Survival curves of patients with HCC according to the background liver disease are shown in Figure 2. During the median follow-up period of 19 months, 349 of 622 patients died (56.1%), of whom 31 were NAFLD-related HCC patients (55.4% of NAFLD-related HCC patients), 89 were ALD-related HCC patients (51.4% of ALD-related HCC), and 229 were HBV-related HCC patients (58.3% of HBV-related HCC). The cumulative probabilities of survival at 1 year and 3 years were, respectively, 54% and 34% in NAFLD-related HCC patients versus 67% and 43% in ALD-related HCC patients, and 57% and 38% in HBV-related HCC patients. The median survival was 14.0 months (95% CI, 1.6–26.4) in NAFLD-related HCC patients, 27.0 months (95% CI, 18.0–36.0) in ALD-related HCC patients, and 17.0 months (95% CI, 11.8–22.2) in HBV-related HCC patients. There was no significant difference in survival among the three groups (*P*=0.135).

### 3.4. Propensity Score Matching Analysis

**Figure 3:**
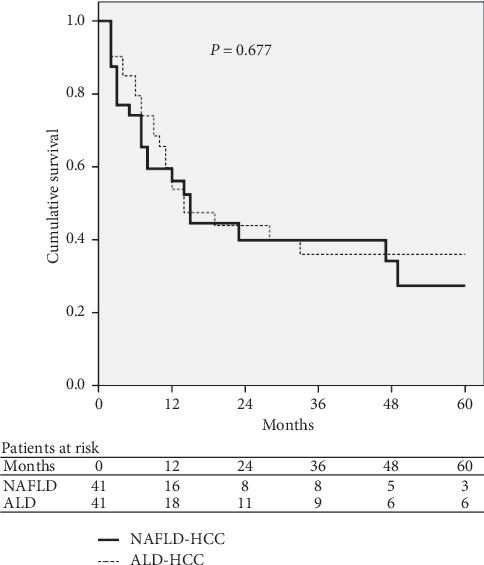
Survival curves of NAFLD-related and ALD-related HCC patients after propensity score matching analysis. ALD: alcoholic liver disease; HCC: hepatocellular carcinoma; NAFLD: nonalcoholic fatty liver disease.

**Figure 4:**
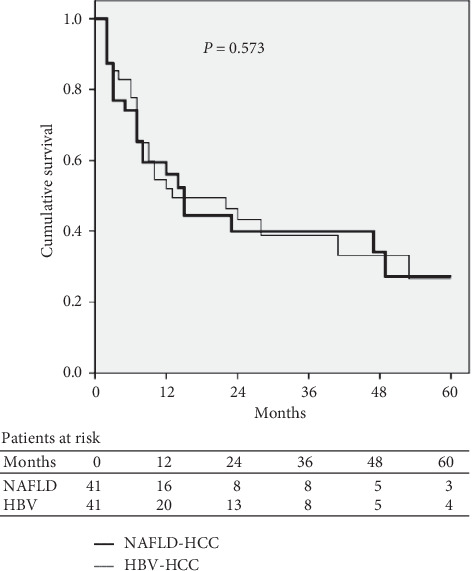
Survival curves of NAFLD-related and HBV-related HCC patients after propensity score matching analysis. HBV: hepatitis B virus; HCC: hepatocellular carcinoma; NAFLD: nonalcoholic fatty liver disease.

The comparison of baseline characteristics of NAFLD-related and ALD-related HCC patients after propensity score matching analysis is shown in Table 4. The cumulative probabilities of survival at 1 year and 3 years were 56% and 40% in NAFLD-related HCC patients versus 55% and 37% in ALD-related HCC patients. The median survival was 14.0 months (95% CI, 2.0–26.0) in NAFLD-related HCC patients and 13.0 months (95% CI, 0–26.3) in ALD-related HCC patients. No significant difference in survival was noted between the two groups (*P*=0.677; Figure 3). Similarly, there were no significant differences in the cumulative probabilities of survival at 1 year and 3 years (56% and 40% versus 53% and 40%) and the median survival (14.0 months [95% CI, 2.0–26.0] vs 12.0 months [95% CI, 4.3–17.8]; *P*=0.573; Figure 4), after propensity score matching analysis between NAFLD-related and HBV-related HCC patients (Table 5).

## 4. Discussion

NAFLD is the most common chronic liver disease in developed societies and its prevalence is increasing rapidly [7, 23, 24]. Most individuals with NAFLD have steatosis, which can develop progressive diseases, including steatohepatitis, cirrhosis, and HCC [14, 25]. By this time, several studies have compared the clinical features and outcomes between NAFLD-related and HCV-related HCC patients owing to the similarity in their natural history [15, 26–28]. However, a comparison of NAFLD-related HCC and ALD-related or HBV-related HCC has not been satisfactorily performed.

In this retrospective study, NAFLD-related HCC patients were more commonly women and had metabolic risk factors more often, including diabetes mellitus and hypertension, compared to ALD-related or HBV-related HCC patients. This result is similar to that observed in previous studies about NAFLD-related and HCV-related HCC patients [13, 15, 23]. Moreover, NAFLD is a hepatic manifestation of metabolic syndrome, which is more often observed in women and is often associated with the presence of insulin resistance and type 2 diabetes mellitus [9, 29, 30].

In the present study, we found that tumor characteristics showed significant differences among the three groups. NAFLD-related HCCs were more often detected at an advanced tumor stage with an infiltrative pattern compared to ALD-related or HBV-related HCCs, consistent with previous results [15, 23]. NAFLD-related HCCs were usually larger, exceeded the Milan criteria, and were more frequently BCLC stage C, but were diagnosed less frequently during surveillance. In addition, NAFLD-related HCC patients were older at HCC diagnosis, had cirrhosis and ascites less often, and had better liver function compared to ALD-related or HBV-related HCC patients. These results should be used to make a delayed diagnosis of NAFLD-related HCC owing to the absence of recognized risk factors such as cirrhosis with proper liver function. In general, cirrhotic patients with NAFLD undergo screening per currently recommended guidelines because the presence of cirrhosis results in a much higher risk of HCC similar to other etiologies [25]. However, not having cirrhosis or having cryptogenic cirrhosis with HCC may be more common in NAFLD than in other etiologies and it is less likely to be detected during liver cancer surveillance, resulting in a delayed diagnosis.

Although NAFLD-related HCC patients were more often detected at an advanced tumor stage due to delayed diagnosis, more patients with NAFLD-related HCC were eligible for liver resection compared to those in the other two groups. This might be because their liver function measurements, such as Child-Pugh score and MELD score, were preserved to a higher extent compared to the scores in the other two groups.

The present study demonstrated that the survival of NAFLD-related HCC patients was similar to that of ALD-related or HBV-related HCC patients. Several confounders with known impact on survival were significantly different among three groups. When confounders were eliminated using propensity score matching analysis, the survival also showed no significant difference between NAFLD-related and ALD-related or HBV-related HCC patients. NAFLD-related HCC showed two conflicting aspects that affect the survival of patients. The favorable aspects were the absence of potential risk factors, less common accompanying liver cirrhosis with ascites, and relatively better liver function considering the Child-Pugh score and MELD score. In contrast, the unfavorable aspects were older age, irregular tumor surveillance, more advanced tumor and clinical-stage with tumor size, vascular invasion, infiltrative pattern, Milan criteria, and BCLC staging. These aspects are likely to make an explanation for the similar overall survival observed among the patients with NAFLD-related, ALD-related, or HBV-related HCC.

The current study has several limitations. First, the retrospective nature of the study design may make it difficult to generalize our findings owing to selection or information bias. Especially, as Korea is known as the HBV endemic area, a portion of patients with isolated anti-HBc may have occult HBV infection affecting the progression of liver diseases to liver cirrhosis and HCC. Because serostatus of anti-HBc was not available in some cases of HCC in this retrospective study, occult HBV infection would be likely ignored in NAFLD-related and ALD-related HCC patients. Second, owing to the different clinical settings in NAFLD, ALD, and HBV patients, several baseline variables could not be sufficiently taken into account through a propensity score model. However, we tried to maximally reduce and adjust a clinical bias for all the baseline variables using propensity score analysis with the nearest-neighbor manner-based one-by-one matching. Third, this is a single-center study that may not reflect the diversity and availability of treatment modality by other medical institutions, being also the main factor to affect the survival of patients. Fourth, the lead-time bias caused by the time between early diagnosis with screening and the time at which diagnosis would have been made without screening [15] could have an effect on the interpretation of a 5-year survival rate. Therefore, further prospective studies are warranted after correcting the lead-time bias for confirming our findings.

## 5. Conclusions

We conclude that NAFLD-related HCCs were more often detected at an advanced tumor stage with infiltrative patterns, although they showed no significant difference in survival compared to ALD-related or HBV-related HCCs. Nevertheless, a strict surveillance program with better screening modalities is required for the early detection and timely treatment of HCC in patients with NAFLD. A future prospective research should be focused on identifying NAFLD patients who require strict surveillance.

## Figures and Tables

**Table 1:** Baseline demographic and clinical characteristics of patients.

Variables	HCC on NAFLD (*n* = 56)	HCC on ALD (*n* = 173)	HCC on HBV (*n* = 393)	*P* value
Demographic and clinical				
Age (years), mean (SD)	68.0 (10.9)	64.1 (9.4)	56.2 (10.4)	<0.001
Male gender, *n* (%)	35 (62.5%)	167 (96.5%)	312 (79.4%)	<0.001
Body mass index (kg/m^2^), mean (SD)	22.7 (6.7)	22.6 (6.5)	22.4 (6.5)	0.860
Tobacco, *n* (%)	17 (30.4%)	87 (50.3%)	137 (34.9%)	0.001
Metabolic risk factors				
Diabetes, *n* (%)	27 (48.2%)	58 (33.5%)	77 (19.6%)	<0.001
Hypertension, *n* (%)	24 (42.9%)	47 (27.2%)	86 (21.9%)	0.003
Blood glucose (mg/dL), mean (SD)	151.5 (67.5)	133.5 (50.8)	126.0 (55.6)	0.005
LDL cholesterol (mg/dL), mean (SD)	89.1 (50.4)	83.1 (54.5)	91.5 (51.1)	0.202
HDL cholesterol (mg/dL), mean (SD)	39.5 (14.5)	40.8 (16.9)	42.1 (18.6)	0.594
Triglycerides (mg/dL), mean (SD)	101.6 (48.3)	87.8 (42.7)	92.1 (54.8)	0.322
Liver function				
Bilirubin (mg/dL), mean (SD)	1.1 (0.9)	2.1 (2.7)	3.0 (7.9)	0.720
Albumin (mg/dL), mean (SD)	3.6 (0.6)	3.3 (0.7)	3.4 (0.7)	0.003
INR, mean (SD)	1.1 (0.4)	1.2 (0.6)	1.3 (1.0)	0.549
Liver cirrhosis, *n* (%)	42 (75.0%)	161 (93.1%)	356 (90.6%)	<0.001
Hepatic encephalopathy, *n* (%)	0 (0.0%)	5 (2.9%)	11 (2.8%)	0.443
Ascites, *n* (%)	18 (32.1%)	97 (56.1%)	185 (47.1%)	0.006
CTP score, mean (SD)	6.2 (1.4)	6.9 (1.6)	6.7 (1.8)	0.014
CTP class A, *n* (%)	39 (69.6%)	82 (47.4%)	215 (54.7%)	0.013
CTP class B, *n* (%)	16 (28.6%)	74 (42.8%)	145 (36.9%)	0.136
CTP class C, *n* (%)	1 (1.8%)	17 (9.8%)	33 (8.4%)	0.158
MELD score, mean (SD)	9.1 (4.1)	12.0 (7.3)	11.3 (6.9)	0.021

HCC: hepatocellular carcinoma; NAFLD: nonalcoholic fatty liver disease; ALD: alcoholic liver disease; HBV: hepatitis B virus; SD: standard deviation; LDL: low-density lipoprotein; HDL: high-density lipoprotein; INR: international normalized ratio; CTP: Child-Turcotte-Pugh; MELD: model for end-stage liver disease.

**Table 2:** Tumor characteristics of patients.

Variables	HCC on NAFLD (*n* = 56)	HCC on ALD (*n* = 173)	HCC on HBV (*n* = 393)	*P* value
Modality of initial tumor detection				
Surveillance, *n* (%)	43 (76.8%)	148 (85.5%)	352 (89.6%)	0.025
Incidental, *n* (%)	6 (10.7%)	7 (4.0%)	6 (1.5%)	0.001
Symptomatic, *n* (%)	7 (12.5%)	18 (10.4%)	35 (8.9%)	0.643
Size of largest tumor (cm), mean (SD)	6.2 (3.4)	3.7 (3.6)	4.5 (4.0)	0.001
Number of nodules, mean (SD)	2.1 (1.6)	2.2 (1.6)	2.1 (1.6)	0.944
Milan out, *n* (%)	35 (62.5%)	62 (35.8%)	184 (46.8%)	0.001
Barcelona clinic liver cancer				
Stage 0, *n* (%)	3 (5.4%)	24 (13.9%)	70 (17.8%)	0.043
Stage A, *n* (%)	18 (32.1%)	76 (43.9%)	128 (32.6%)	0.029
Stage B, *n* (%)	12 (21.4%)	28 (16.2%)	68 (17.3%)	0.666
Stage C, *n* (%)	20 (35.7%)	22 (12.7%)	91 (23.2%)	<0.001
Stage *D*, *n* (%)	3 (5.4%)	23 (13.3%)	36 (9.2%)	0.154
Infiltrative, *n* (%)	15 (26.8%)	23 (13.3%)	59 (15.0%)	0.047
Extrahepatic metastasis, *n* (%)	17 (30.4%)	35 (20.2%)	108 (27.5%)	0.136
Lymphatic node metastasis, *n* (%)	6 (10.7%)	13 (7.5%)	44 (11.2%)	0.404
Macrovascular infiltration, *n* (%)	17 (30.4%)	33 (19.1%)	118 (30.0%)	0.022
*α*-FP (ng/dL), median (range)	182.0 (1–137648)	26.8 (0.2–150000)	70.5 (0–150000)	<0.001
PIVKAII (mAU/ml), median (range)	654.5 (5–199000)	94.5 (2–457000)	45.5 (6–230642)	0.003

HCC: hepatocellular carcinoma; NAFLD: nonalcoholic fatty liver disease; ALD: alcoholic liver disease; HBV: hepatitis B virus; SD: standard deviation; *α*-FP: alpha-fetoprotein; PIVKAII: protein induced by vitamin K absence or antagonist-II.

**Table 3:** Treatment strategies of patients.

Initial treatment modality	HCC on NAFLD (*n* = 56)	HCC on ALD (*n* = 173)	HCC on HBV (*n* = 393)	*P* value
TACE, *n* (%)	28 (50.0%)	112 (64.7%)	216 (55%)	0.027
Surgical resection, *n* (%)	11 (19.6%)	12 (6.9%)	56 (14.2%)	0.014
RFA, *n* (%)	0 (0.0%)	13 (7.5%)	23 (5.9%)	0.111
Sorafenib, *n* (%)	9 (16.1%)	6 (3.5%)	37 (9.4%)	0.006
Best supportive care, *n* (%)	8 (14.3%)	30 (17.3%)	61 (15.5%)	0.811

HCC: hepatocellular carcinoma; NAFLD: nonalcoholic fatty liver disease; ALD: alcoholic liver disease; HBV: hepatitis B virus; TACE: transcatheter arterial chemoembolization; RFA: radiofrequency ablation.

**Table 4:** Baseline characteristics of NAFLD-related and ALD-related HCC patients after a propensity score match analysis.

Variables	HCC on NAFLD (*n* = 41)	HCC on ALD (*n* = 41)	*P* value
Demographic and clinical			
Age in years, mean (SD)	67.4 (10.7)	65.6 (9.6)	0.289
Male gender, *n* (%)	26 (63.4%)	37 (90.2%)	0.051
Diabetes, *n* (%)	16 (39.0%)	13 (31.7%)	0.488
Ischemic cardiomyopathy, *n* (%)	4 (9.8%)	1 (2.4%)	0.166
Liver function			
CTP class A, *n* (%)	33 (80.5%)	35 (85.4%)	0.557
CTP class B, *n* (%)	8 (19.5%)	6 (14.6%)	0.557
CTP class C, *n* (%)	0 (0.0%)	0 (0.0%)	—
Tumor characteristics			
Size			
Largest nodule (cm), mean (SD)	6.3 (3.4)	6.3 (3.7)	0.256
<2 cm, *n* (%)	4 (9.8%)	3 (7.3%)	0.639
2.1–3 cm, *n* (%)	2 (4.9%)	4 (9.8%)	0.396
3.1–5 cm, *n* (%)	12 (29.3%)	14 (34.1%)	0.635
>5 cm, *n* (%)	23 (56.1%)	20 (48.8%)	0.507
Number of nodules			
1, *n* (%)	26 (63.4%)	28 (68.3%)	0.641
2–3, *n* (%)	6 (14.6%)	6 (14.6%)	1.000
>3, *n* (%)	9 (22.0%)	7 (17.1%)	0.577
Infiltrative, *n* (%)	0 (0.0%)	0 (0.0%)	—
Milan out, *n* (%)	26 (63.4%)	23 (56.1%)	0.499
Macrovascular infiltration, *n* (%)	8 (19.5%)	6 (14.6%)	0.557
Detection on surveillance, *n* (%)	31 (75.6%)	35 (85.4%)	0.265
Initial treatment modality			
TACE, *n* (%)	21 (51.2%)	25 (61.0%)	0.267
Surgical resection, *n* (%)	11 (26.8%)	6 (14.6%)	0.173
RFA, *n* (%)	0 (0.0%)	1 (2.4%)	0.314
Sorafenib, *n* (%)	3 (7.3%)	2 (4.9%)	0.644
Best supportive care, *n* (%)	6 (14.6%)	7 (17.1%)	0.762

HCC: hepatocellular carcinoma; NAFLD: nonalcoholic fatty liver disease; ALD: alcoholic liver disease; SD: standard deviation; CTP: Child-Turcotte-Pugh; TACE: transcatheter arterial chemoembolization; RFA: radiofrequency ablation.

**Table 5:** Baseline characteristics of NAFLD-related and HBV-related HCC patients after a propensity score match analysis.

Variables	HCC on NAFLD (*n* = 41)	HCC on HBV (*n* = 41)	*P* value
Demographic and clinical			
Age in years, mean (SD)	67.4 (10.8)	66.7 (11.1)	0.854
Male gender, *n* (%)	26 (63.4%)	26 (63.4%)	1.000
Diabetes, *n* (%)	16 (39.0%)	12 (29.3%)	0.352
Ischemic cardiomyopathy, *n* (%)	4 (9.8%)	3 (7.3%)	0.693
Liver function			
CTP class A, *n* (%)	33 (80.5%)	34 (82.9%)	0.775
CTP class B, *n* (%)	8 (19.5%)	7 (17.1%)	0.775
CTP class C, *n* (%)	0 (0.0%)	0 (0.0%)	—
Tumor characteristics			
Size			
Largest nodule (cm), mean (SD)	6.3 (3.4)	7.1 (4.6)	0.082
<2 cm, *n* (%)	4 (9.8%)	6 (14.6%)	0.500
2.1–3 cm, *n* (%)	2 (4.9%)	3 (7.3%)	0.644
3.1–5 cm, *n* (%)	12 (29.3%)	7 (17.1%)	0.191
>5 cm, *n* (%)	23 (56.1%)	25 (61.0%)	0.654
Number of nodules			
1, *n* (%)	26 (63.4%)	27 (65.9%)	0.817
2–3, *n* (%)	6 (14.6%)	5 (12.2%)	0.746
>3, *n* (%)	9 (22.0%)	9 (22.0%)	1.000
Infiltrative, *n* (%)	0 (0.0%)	0 (0.0%)	—
Milan out, *n* (%)	26 (63.4%)	25 (61.0%)	0.820
Macrovascular infiltration, *n* (%)	8 (19.5%)	9 (22.0%)	0.785
Detection on surveillance, *n* (%)	31 (75.6%)	36 (87.8%)	0.153
Initial treatment modality			
TACE, *n* (%)	21 (51.2%)	19 (46.3%)	0.825
Surgical resection, *n* (%)	11 (26.8%)	10 (24.4%)	0.800
RFA, *n* (%)	0 (0.0%)	2 (4.9%)	0.152
Sorafenib, *n* (%)	3 (7.3%)	4 (9.8%)	0.693
Best supportive care, *n* (%)	6 (15.0%)	6 (14.6%)	1.000

HCC: hepatocellular carcinoma; NAFLD: nonalcoholic fatty liver disease; HBV: hepatitis B virus; SD: standard deviation; CTP: Child-Turcotte-Pugh; TACE: transcatheter arterial chemoembolization; RFA: radiofrequency ablation.

## Data Availability

The data used to support the findings of this study are available from the corresponding author upon request.
